# Thermal perception thresholds among workers in a cold climate

**DOI:** 10.1007/s00420-017-1227-x

**Published:** 2017-05-11

**Authors:** Lage Burström, Bodil Björ, Tohr Nilsson, Hans Pettersson, Ingemar Rödin, Jens Wahlström

**Affiliations:** 1 Department of Public Health and Clinical Medicine, Occupational and Environmental Medicine, Umeå University, 901 87 Umeå, Sweden; 2 Arcum, Umeå University, Umeå, Sweden; 3 Department of Occupational and Environmental Medicine, University Hospital of North Norway, Tromsø, Norway; 4 Swedish Work Environment Authority, Stockholm, Sweden

**Keywords:** Neurosensory function, Sensory threshold, Normative values, Cold temperature, Mine work

## Abstract

**Purpose:**

To investigate whether exposure to cold could influence the thermal perception thresholds in a working population.

**Methods:**

This cross-sectional study was comprised of 251 males and females and was carried out at two mines in the northern part of Norway and Sweden. The testing included a baseline questionnaire, a clinical examination and measurements of thermal perception thresholds, on both hands, the index (Digit 2) and little (Digit 5) fingers, for heat and cold.

**Results:**

The thermal perception thresholds were affected by age, gender and test site. The thresholds were impaired by experiences of frostbite in the fingers and the use of medication that potentially could affect neurosensory functions. No differences were found between the calculated normative values for these workers and those in other comparative investigations conducted in warmer climates.

**Conclusions:**

The study provided no support for the hypothesis that living and working in cold climate will lead to impaired thermal perception thresholds. Exposure to cold that had caused localized damage in the form of frostbite was shown to lead to impaired thermal perception.

## Introduction

Disturbance of the thermal perception occurs in many neurological patients related to, for instance, diabetes, chemotherapy, amyloidosis or immunological causes, as a consequence of lesions in the peripheral or central nervous system (Callaghan et al. 2015; Chan and Wilder-Smith 2016; Fruhstorfer et al. 1976; Guy et al. 1985; Heldestad and Nordh 2007). There are also work related exposures associated with thermal perception deterioration such as cold, vibration, and mechanical stress (Nilsson and Lundström 2001). The influence related to exposure to vibration and mechanical stress have been widely studied [see for instance (Rolke et al. 2013; Toibana et al. 2000)] but the influence of cold on the thermal perception has rarely been investigated. Carlsson et al. (2016) found that 14 months of military training comprising cold winter conditions reduced sensation from heat and cold in the hands. Participants who reported a cold injury had a more pronounced reduction in their thermal sensitivity. Such reduction in thermal perception after local freezing cold injury has also been shown in a previous study (Carlsson et al. 2014).

Thermal perception threshold testing is a psychophysics test used for monitoring and early detection of reduced thermal perception (Bakkers et al. 2013; Gerr and Letz 1994; Hafner et al. 2015; Rolke et al. 2006a, b). The test evaluates the peripheral small fibre function, as heat experience is mediated by thin unmyelinated fibres (C-fibres) and the corresponding fibres mediating cool sensations are thin myelinated peripheral fibres (A-δ) (Purves et al. 2012). The mediated stimuli information is transferred to the central nervous system, where the information is evaluated. However, the results of the tests could be influenced by several technical factors such as the devices and test settings used, as well as individual variables such as age, gender and anthropometric data (Bakkers et al. 2013).

Since the northern part of the Nordic countries is characterized by cold climate where frostbite is common (Stjernbrandt et al. 2017), the aim was to investigate whether prolonged exposure to cold influences the thermal perception thresholds in a population living and working in a cold climate.

## Methods

### Participants

The study group consisted of employees at mining companies in the northern part of Sweden and Norway, where the average annual ambient temperature is about ±1.0 °C. In January, the average ambient temperature is about −14 °C in the Swedish mine and about −9 °C in the Norwegian mine. Out of a total 485 workers listed on the employee rosters, 251 (51.7%) agreed to participate. The group of mine workers included in the study represents all the different occupations at the mines, with the main groups being vehicle drivers, mechanics and electricians. When being invited to participate, the participants were informed about the study both verbally and in writing and they gave their written consent. The Regional Ethical Review Board for medical research in Umeå, Sweden (2012-365-31M), as well as in Oslo, Norway (2013/1026/REK), approved the study.

### Experimental procedure

The measurements of the thermal perception thresholds were all carried out by the same two experienced test leaders in a quiet room with a temperature of 22 °C (±1 °C) and with an air flow not noticeable, i.e., below 0.2 m/s (Lindsell and Griffin 2002). The participants were acclimatized for at least 15 min followed by approximately 15 min of testing (Griffin and Bovenzi 2007). Cold and hot thermal thresholds were determined using Modular Sensory Analyser (MSA Thermotest, Somedic AB), using a rectangular 2.5 × 5.0 cm stimulation probe of Peltier-type, and the instrument set-up is calibrated annually. The test sites were distal phalanges on the right and left hand, index (Digit 2) and little (Digit 5) finger and the forearm was supported on a bench. The test area covered approximately 45 mm of the fingers length, respectively.

Prior to testing, finger skin temperature was measured by infrared thermometer (TESTO 845) and, if lower than 28 °C, hands were heated using warm running water until the temperature of the dried fingers was above 28 °C. The duration of heating with water was not long enough to make the skin of the fingers could swell. Scripted verbal instructions were used to administer the test. Before testing, the participant was instructed to firmly but in a relaxed way apply the tested area on top of the stimulation probe. No strapping of the probe to the test site was applied. The participant was instructed to press an electrical switch as soon as they perceived an alteration in thermal sensation.

A reaction time-dependent method, the ‘method of limits’ was used for testing (Hilz et al. 1999), with a starting (baseline) temperature of 32.0 °C. The rate of temperature change was 1 °C/s, with a return to baseline temperature at 3 °C/s, within the range of 10–52 °C. Thermal testing was done by a series of 5 consecutive cold stimuli followed by 5 warm stimuli for each test site. The inter-stimulus time varied between 2 and 6 s, and the thermal thresholds at the site measured was defined as the average of the individual cold and heat threshold reported by the participant for each stimulus.

The participants were medically examined by a physician and completed questionnaires that covered basic information such as age, sex, weight, height, smoking and use of snuff. It also covered workers’ perceived experiences of work and health, their use of hand-held vibrating tools and how many hours in a typical working day they were outdoors or in an unheated building/machine. General health was measured on the basis of the question: “How would you rate your general health?” on a category scale of Very good, Good, OK, Bad and Very bad. In the medical examination, the physician noted the retrospective medical history and performed a physical examination to identify possible medical conditions that could affect the results, such as use of medication, completed medical treatments, diabetes and experience of frostbite.

### Statistics

The thermal perception thresholds are presented as the temperature change in degrees Celsius from the baseline temperature to the detection temperature. The Shapiro–Wilk test and histogram was used for investigating whether the data was normally distributed and none of the measured thermal perception thresholds fulfilled the requirements for normality. Wilcoxon signed-rank and Mann–Whitney tests were used for analysing changes in the non-normal distributed variables.

The questions regarding general health were dichotomised, where the first two answers “Very good and Good” were considered to represent good health and the other three answers were considered to represent not so good health. The miner’s individual list of medication or completed medical treatments was dichotomised into use of medication/treatments that could or could not affect neurosensory functions. Treatment considered to affect neurosensory functions was medication for diabetes and thyroid diseases, high cholesterol and triglycerides, as well medication used for cancer treatment (Chan and Wilder-Smith 2016).

Analyses on difference in background information were carried out using ANOVA. Tobacco users were defined as those using either snuff or smoked. Hours in a typical working day, the participants were outdoors or in an unheated building/machine, were transformed by median split to a categorical variable. All calculations were performed with the statistical program IBM SPSS version 23 (IBM Corp, 2015) and *p* values <0.05 were considered to be statistically significant.

## Results

The characteristics of the participating workers are given in Table 1.

**Table 1:** Characteristics of participating mine workers shown by gender

	All participants	Female	Male
Number of workers	251	75	176
Age (years)	40.6 (12.0)	36.8 (10.7)*	42.2 (12.2)
Height (cm)	174.1 (8.7)	166.0 (6.7)*	177.6 (7.0)
Weight (kg)	82.2 (17.0)	71.5 (17.0)*	86.8 (14.7)
Outdoor work (h)	2.4 (2.7)	1.3 (2.2)*	2.8 (2.7)
Use of tobacco	41.6	36.5	43
Use of vibrating tools	28.3	12.0*	35.2
Not so good self-perceived health	15.3	21.3	12.7
Diabetes	2.8	0.0	4.0
Frostbite in fingers	13.1	10.7	14.2
Medication/treatments that could affect neurosensory functions	11.2	9.3	11.9

Data are given as means (standard deviations) or numbers (%)

* *p* < 0.001

The mean age of participating mine workers was 41 years (Table 1) with a range between 18 and 64 years. The proportion of females in the study was 30%. Statistical analyses showed that there was a significant difference between males and females in terms of age, height, weight, outdoor work and use of handheld tools, but not as regards use of tobacco, rated health, frost bites and use of medication that could affect neurosensory functions. Diabetes was only present among 7 males.

The calculated mean thermal perception thresholds for heat and cold for the different test sites are shown in Table 2, stratified by gender.

**Table 2:** Thermal perception detection thresholds (heat and cold) presented as the mean difference in degrees Celsius (°C) from baseline temperature of 32 °C to detection temperature [95% CI = 95% confidence interval], stratified by gender

		All participants		Female		Male	
		Mean	95% CI	Mean	95% CI	Mean	95% CI
Heat							
Digit 2							
Right		3.5	3.2–3.8	2.9	2.5–3.3	3.8	3.4–4.1
Left		3.3	3.1–3.5	2.7	2.4–3.0	3.5	3.3–3.8
Digit 5							
Right		4.7	4.4–4.9	4.2	3.7–4.7	4.9	4.5–5.2
Left		4.8	4.5–5.1	4.0	3.5–4.5	5.2	4.8–5.5
Cold							
Digit 2							
Right		2.9	2.7–3.1	2.7	2.4–2.9	3.0	2.8–3.2
Left		2.5	2.4–2.6	2.3	2.0–2.6	2.6	2.4–2.8
Digit 5							
Right		3.4	3.2–3.6	3.3	2.9–3.7	3.5	3.2–3.7
Left		3.5	3.2–3.7	3.2	2.8–3.5	3.6	3.3–3.9

The results show that the threshold averages for all participants vary between 2.5 and 4.8 °C in the different test sites. Statistical analysis shows that there was a significant difference (*p* < 0.001) between Digit 2 and Digit 5 for all thresholds. No differences in thresholds for hand-side were found (0.172 < *p* < 0.537), except for Digit 2 cold threshold (*p* < 0.001). There was a significant difference (0.001 < *p* < 0.006) between male and female participants for all test sites but not for Digit 5, cold thresholds (0.243 < *p* < 0.294). Use of handheld tools, smoking habits and perceived health had no significant influence on measured thresholds.

There was a statistical significant (0.001 < *p* < 0.032) reduced sensitivity in measured heat and cold thresholds when stratifying by age, Table 3. The older participants (above 40 years) had a reduced sensitivity in the range of 0–0.6 °C. The same was found for participants who had above average body weight in heat thresholds (0.006 < *p* < 0.021), but not in cold thresholds (0.200 < *p* < 0.757). Height, use of vibrating tools and working outdoor had no significant influence on measured thresholds.

**Table 3:** Thermal perception detection thresholds (heat and cold) presented as the mean difference in degrees Celsius (°C) from baseline temperature of 32 °C to detection temperature [95% CI = 95% confidence interval], stratified by age

		All participants (*n* = 251)		Participants under or equal to 40 years (*n* = 121)		Participants above 40 years (*n* = 130)	
		Mean	95% CI	Mean	95% CI	Mean	95% CI
Heat							
Digit 2							
Right		3.5	3.2–3.8	3.1*	2.8–3.5	3.9	3.5–4.2
Left		3.3	3.1–3.5	3.0*	2.7–3.2	3.6	3.3–3.9
Digit 5							
Right		4.7	4.4–4.9	4.2*	3.8–4.6	5.1	4.7–5.5
Left		4.8	4.5–5.1	4.4*	4.0–4.8	5.2	4.8–5.7
Cold							
Digit 2							
Right		2.9	2.7–3.1	2.7*	2.5–2.9	3.1	2.8–3.4
Left		2.5	2.4–2.6	2.3*	2.1–2.4	2.7	2.5–3.0
Digit 5							
Right		3.4	3.2–3.6	3.1*	2.9–3.4	3.7	3.4–4.0
Left		3.5	3.2–3.7	3.1*	2.8–3.4	3.8	3.4–4.2

* *p* < 0.01

Table 4 provides the calculated mean thermal perception thresholds for heat and cold for participants (*n* = 193) not affected by frostbite in the fingers or use of medication/treatments. In the table is also given the corresponding thresholds for participants that have had frostbite in the fingers (*n* = 33) as well as for the participants that used medication/treatments that could affect neurosensory functions (*n* = 28; 3 participants also had frostbite). Experience of frostbite in the fingers increased the mean thresholds with 0.1–0.8 °C for all sites compared to non-affected participants and was significant for heat threshold Digit 2 (0.013 < *p* < 0.039). Diabetes as diagnose did not influence the thresholds, but use of medication/treatments that could affect neurosensory functions, increased the mean thresholds for all sites (range 0.2–1.1 °C) compared to non-affected participants and had a significant influence on both heat and cold thresholds for all but three sites (Heat Digit 5 Left, Cold Digit 2 Right/Left).

**Table 4:** Thermal perception detection thresholds (heat and cold) presented as the mean difference in degrees Celsius (°C) from baseline temperature of 32 °C to detection temperature [95% CI = 95% confidence interval], stratified by normal participants, participants with frostbite in fingers or participant with use of affecting medication/treatments

		Normal participants (*n* = 193)		Participants with frostbite in fingers (*n* = 33)		Participant with use of affecting medication/treatments (*n* = 28)	
		Mean	95% CI	Mean	95% CI	Mean	95% CI
Heat							
Digit 2							
Right		3.3	3.0–3.6	4.2*	3.4–5.0	4.3*	3.4–5.2
Left		3.1	2.9–3.4	3.8*	3.2–4.5	3.9**	3.2–4.6
Digit 5							
Right		4.5	4.2–4.8	4.9	4.4–5.5	5.7*	4.8–6.5
Left		4.7	4.3–5.0	5.2	4.4–6.0	5.2	4.3–6.1
Cold							
Digit 2							
Right		2.9	2.7–3.1	3.0	2.5–3.5	2.8	2.4–3.1
Left		2.4	2.3–2.6	2.8	2.3–3.3	2.7	2.3–3.0
Digit 5							
Right		3.3	3.1–3.5	3.6	3.0–4.2	4.1*	3.3–4.8
Left		3.4	3.1–3.6	3.8	2.9–4.8	3.8*	3.2–4.3

* *p* < 0.05; ** *p* < 0.01

## Discussion

Our results show that exposure to cold that causes localized damage in the form of frostbites (Beise et al. 1998) leads to impaired thermal perception which is in line with the results of previous studies (Carlsson et al. 2014, 2016).

**Fig. 1:**
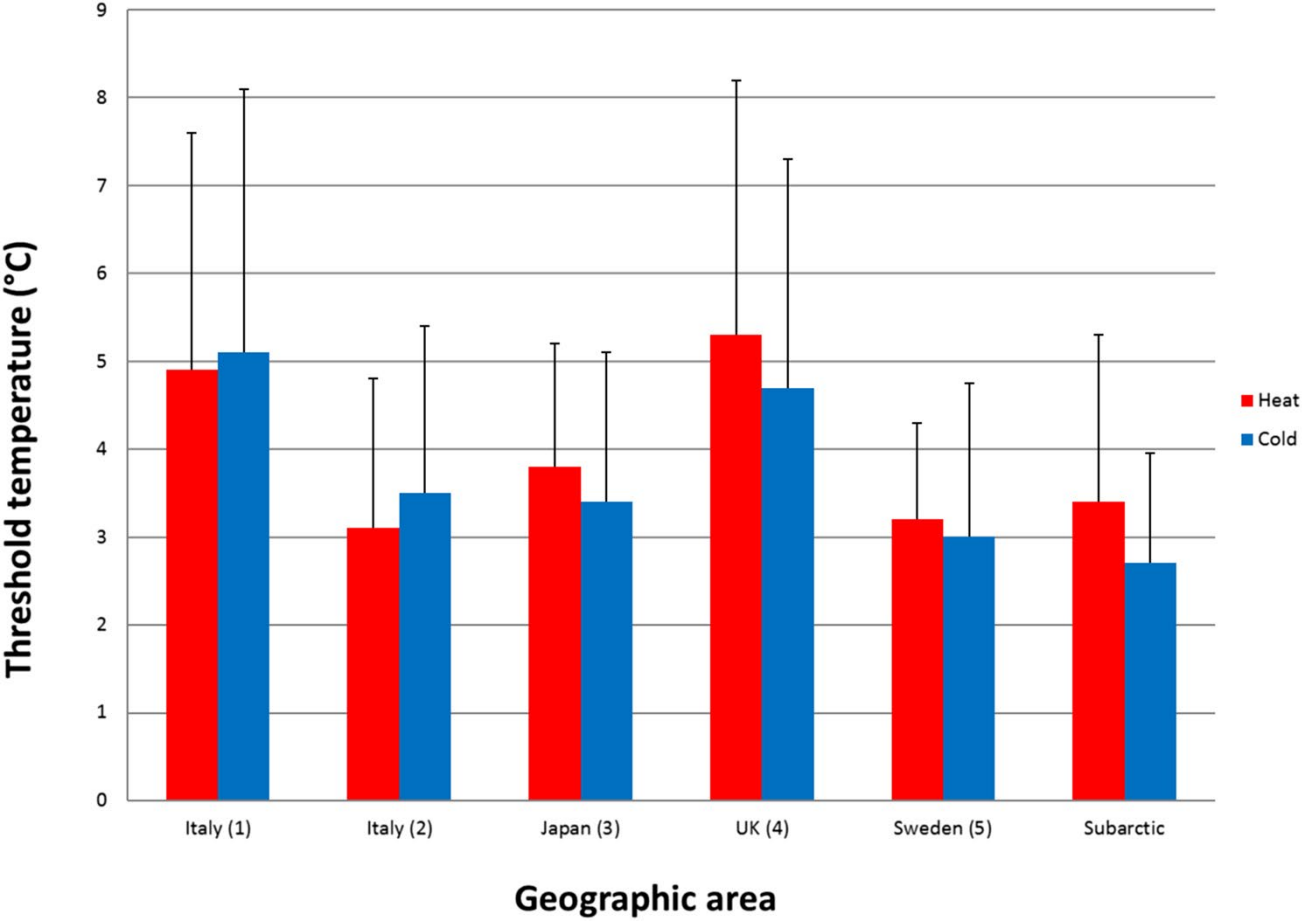
Comparison of the measured mean difference in degrees Celsius (°C) from baseline temperature to detection temperature in different geographic areas with standard deviation* bars*. The annual ambient temperature for each region and the number of participants in each study are 1 (Bovenzi et al. 2011) *n* = 63, 14 °C, 2 (Miscio et al. 2005) *n* = 51, 12 °C, 3 (Sakakibara et al. 2002) *n* = 56, 11 °C, 4 (Seah and Griffin 2008) *n* = 80, 10 °C, 5 (Carlsson et al. 2016) *n* = 81, 0 °C

Comparing our measured thermal perception thresholds with the ones in earlier publications is difficult since the results are dependent on the investigators choices of psycho-physical method, tested modality, design, and instrumentation (Nilsson et al. 2008). Moreover, in other studies conducted, the authors have sometimes included all participants [representative selection like (Gerr and Letz 1994)] or at other times just healthy participants. Healthy participants could either be selected from “subjective health”, where inclusion requires the participant’s subjective experience of good health [for example (Heldestad et al. 2010; Lindsell and Griffin 2002; Seah and Griffin 2008)], or by “objective health”, where the participant also needs to undergo a medical examination so as to rule out diseases such as diabetes, use of medication or previous experiences of frostbites, which could affect the results (Carlsson et al. 2014; Gierthmuhlen et al. 2015; Nilsson et al. 2008). In our study, we used the latter and 23% (58 out of 251) of the participants were excluded due to the use of medication/treatments or previous experiences of frostbites for establishing normative values. Reports on normative values differ considerably (Bakkers et al. 2013), which makes possible comparisons with previous studies of magnitude, abnormality and normative values uncertain. However, for comparison, the results of studies in different geographic regions (Bovenzi et al. 2011; Carlsson et al. 2016; Miscio et al. 2005; Sakakibara et al. 2002; Seah and Griffin 2008) have been summarized in Fig. 1 together with the results from the subarctic region obtained in our study. The comparison applies to the index finger and the measured thermal perception thresholds for heat and cold, respectively.

Visually comparing our results with the studies earlier presented, shows similar findings of mean value and deviation. Our results, therefore, do not provide any evidence to suggest an association between exposure to cold climate and impaired thermal perception thresholds among workers in the Nordic sub-arctic area.

The results in our material show that age and gender affect thermal perception thresholds, where males generally have reduced perception compared to females and persons older than 40 years generally have a reduced thermal perception compared to persons younger than 40 years. The changes in absolute values are quite small, although significant. These results are in line with previous published results (Bakkers et al. 2013; Gerr and Letz 1994; Gonzalez-Duarte et al. 2016; Heldestad and Nordh 2007; Lindsell and Griffin 2002; Liou et al. 1999; Nilsson et al. 2008; Rolke et al. 2006b; Seah and Griffin 2008). The gender differences in thermal perception thresholds found in this study, sometimes have been explained by differences in anthropometric data such as weight and height (Nilsson et al. 2008; Torgen and Swerup 2002). Other explanations have been that females are more perceptually sensitive to warmth and cold than males (Bartlett et al. 1998), increased density of thermoreceptors in females and possibly increased spatial summation (Golja et al. 2004) and changes in sensation among females across the menstrual cycle due to hormone changes (Soderberg et al. 2006).

There was a significant difference in measured thresholds between the index and little finger. The results are in accordance with those from Ekenvall et al. (1990). One explanation could be that the number of cold or warm sensory units in any one surface area are so few, that a higher sensitivity for the index finger measurements might be expected than for the little finger measurements, considering the contact area (Nilsson et al. 2008). Our results show that height did not influence the measured thresholds while body weight affected the heat thresholds. In the scientific literature can be found studies that both support and do not support these results [for references see (Heldestad et al. 2010)]. It is worth noticing that we in our study, carried out in a working population, also found that use of medication that could affect neurosensory functions had a significant influence on measured thermal perception thresholds. Use of this type of medication is related to a diagnosed disease and we cannot determine whether it is the medication per se or the underlying disease that causes impaired thresholds.

There could be many sources of a possible bias in our study. The importance of the individual examiner has been stressed repeatedly (Gerr and Letz 1994), but in our study we found no difference in measured mean thresholds between the test leaders. Furthermore, the reaction time-dependent method for detecting the thermal thresholds could also have affected the measures (Levy et al. 1989) as well as the finger push force applied to the stimulation probe (Maeda and Sakakibara 2002). In the cross-sectional design, bias might have arisen through differential healthy worker selection and reduce the association between exposure to cold climate and studied thermal perception thresholds. Workers with “cold sensitivity”, i.e., abnormal reaction to cold exposure, could have quit their jobs. However, since the annual employee turnover is low, we do not believe that such a selection influenced the results. The daily and life time exposure to cold climate may also differ greatly between individual workers. Some of them may have spent hours of their working day outdoor, while others may have worked mainly indoors, in vehicle-cabins, and the time they have spent outdoors is limited. We have no information on the individual total exposure to cold that include both work and leisure time activities. Moreover, we have no information on the participants use of protective clothing during both work and leisure time. The influence of frostbite that we observed could have been influenced on the grade of the cold injury, where we have made no distinction.

## Conclusions

Overall, our findings provided no support for the hypothesis that working and living in a cold climate will influence the thermal perception thresholds. However, exposure to cold that caused localized damage in the form of frostbites was shown to lead to impaired thermal perception, which highlights the need for the use of protective clothing against cold.
